# Correction to “Physiologically‐based pharmacokinetic pharmacodynamic parent‐metabolite model of edoxaban to predict drug–drug‐disease interactions: M4 contribution”

**DOI:** 10.1002/psp4.13187

**Published:** 2024-06-06

Xu R, Liu W, Ge W, He H, Jiang Q. *CPT Pharmacometrics Syst Pharmacol*. 2023;12(8):1093‐1106.

In the title page, the author affiliation “Wenyuan Liu^1,3^ and Weihong Ge^1,3^” was incorrect. This should be changed to “Wenyuan Liu^3^ and Weihong Ge^3.^”

We apologize for this error.

